# Supplementary material for: Co-Administration of High-Dose Nebulized Colistin for Acinetobacter baumannii Bacteremic Ventilator-Associated Pneumonia: Impact on Outcomes
Source: Antibiotics (Basel). 2024 Feb 8;13(2):169. doi: 10.3390/antibiotics13020169 (PMC10886014; doi:10.3390/antibiotics13020169)
Supplement: Supplementary file 1 [file antibiotics-13-00169-s001.zip › antibiotics-2850628-supplementary.pdf]

Supplemental Table S1. Propensity score- adjusted analysis on A. baumannii attributed mortality

parameter	Odds ratio	95% CI (lower – upper limit)	p- value
Propensity score	8.148	0.060- 1109	0.403
Ampicillin /sulbactam administration	0.976	0.291 – 3.273	0.976
Nebulized colistin administration	0.228	0.062 – 0.840	0.026
Pan- drug Resistant (PDR) strain	1.075	0.159 – 7.262	0.941
